# Laser-induced fibers and copper phthalocyanine modified laser-induced graphene electrodes for sensitive and selective electrochemical detection of nitrite†

**DOI:** 10.1039/d4ra03341h

**Published:** 2024-09-09

**Authors:** Anurag Adiraju, Aditya Jalasutram, Ammar Al-Hamry, Malak Talbi, Junfei Wang, Christoph Tegenkamp, Olfa Kanoun

**Affiliations:** a Chair Measurement and Sensor Technology, Department of Electrical Engineering and Information Technology, Chemnitz University of Technology 09107 Chemnitz Germany adiraju.anurag@etit.tu-chemnitz.de; b Analysis of Solid Surfaces, Institute for Physics, Chemnitz University of Technology 09107 Chemnitz Germany

## Abstract

We have recently reported laser-induced fibers (LIF) as a promising nanomaterial that possesses good electrochemical activity and are easily manufacturable. In this paper, for the first time, the application of LIF as functionalization materials on laser-induced graphene (LIG) electrodes for the detection of nitrate is demonstrated. The as-fabricated LIF surfaces on Kapton were extracted by ultrasonication as a dispersion and were used to modify the surface of the LIG electrode. An enhancement in active surface area from 0.669 cm^2^ for bare LIG to 0.83 cm^2^ for LIF-modified LIG was observed. Similarly, the heterogeneous electron transfer rate increased from 0.190 to 0.346 cm s^−1^ for LIF/LIG electrodes. The electrochemical detection of nitrite was achieved by modifying the LIG with a nanocomposite of LIF and copper phthalocyanine (CuPc). The presence of CuPc provided the desired catalytic activity towards the oxidation of nitrite, and the LIF enhanced the electron transfer to the electrode. Such a synergetic combination of the LIF embedded with CuPc enabled reaching a low limit of detection (LoD) of 0.12 μM, a large linear range from 10 to 10 000 μM and good selectivity in the presence of potential interferants. The sensor had a long shelf life of 30 days and good analytical capability to detect nitrite in mineral, tap, and groundwater. The potential of LIF is largely unexplored and the findings reported here on the fibers would open manifold opportunities for realizing novel applications.

## Abstract 2

This work demonstrates the potential of laser-induced fibers as functionalization material for electrochemical sensors and implements copper phthalocyanine and laser-induced fibers modified laser-induced graphene electrodes to detect nitrite.

## Graphical abstract

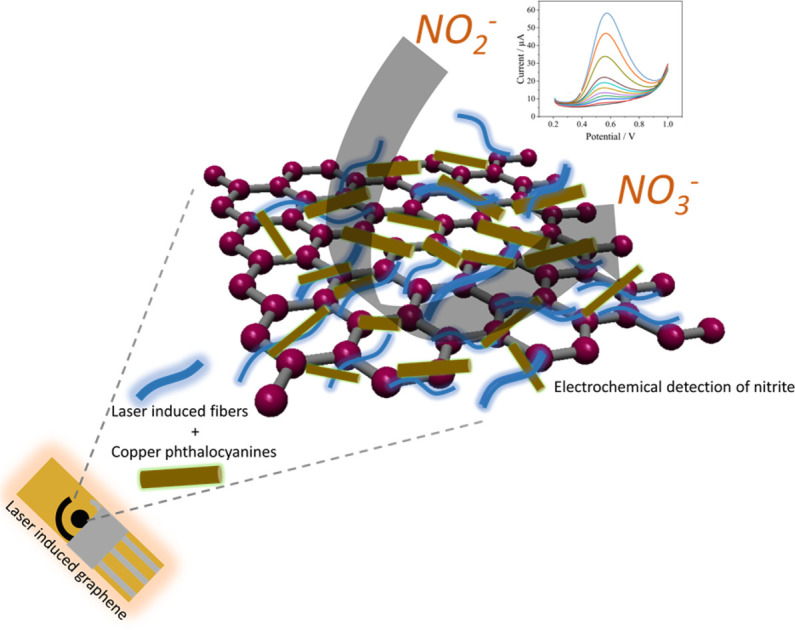

## Introduction

Carbon fibers, with typical dimensions in micrometers, are made of sp^2^-bonded carbon atoms possessing good electrical conductivity, high mechanical strength, high surface area, and superior thermal properties.^1,2^ Owing to their valuable properties, their applications in the field of sensors include structural health monitoring as strain sensors, temperature and humidity sensors,^3^ and modification materials for electrochemical sensors.^4^ Their use in electrochemistry could be related to the fact that they possess good intrinsic carrier mobility, stability, and enhanced electrical conductivity.^5^ In addition, they are also widely used as materials for microelectrodes.^6^ The current production methods of the fibers include thermal, chemical vapor deposition and electrospinning, followed by heat treatment^4^ with different polymer precursors that include polyacrylonitrile, polyvinyl alcohol, and polyimides, to name a few.^7^ This is followed by stabilization in air, carbonization, and a final heat treatment in an inert environment.^8^ However, these methods necessitate long durations, requiring expensive precursor materials and specialized equipment, with a low production rate of fibers.

CO_2_ laser interaction with polyimide sheet such as kapton produces LIG electrodes with high surface area and at certain combinations of laser powers also produces LIF surfaces consisting of protruding fibers from the surface. These fibers have dimensions in the order of nanometers on the surface.^9,10^ Recently, we have reported on the conductive nature of these fibers formed on LIG in a single, cost-effective step by irradiation of CO_2_ laser beam on the polyimide substrate.^10^ As the fibers are loosely bound, extracting these fibers in the form of a dispersion (LIF dispersions) and modifying electrochemical electrodes with them showed an enhancement in electrochemical activity in the presence of standard redox probes. Building on the previous work, this research aims to investigate and demonstrate the usefulness of LIF as a modification material for the electrochemical detection of nitrite, thus proposing an alternative way to produce carbon fibers and exploring the potential prospects of LIF.

Nitrite, an important constituent in the nitrogen cycle, is used as a food preservative,^11^ rust inhibitor,^12^ and fertilizer^13^ for crops. However, nitrite has several detrimental effects on human health as its intake can cause cancer,^14^ blue baby syndrome,^15^ and damage to the central nervous system.^16^ In this regard, the U.S. Environmental Protection Agency has set 21.7 μM as the maximum allowable level for nitrite in the environment^17^ and electrochemical sensors are a promising alternative to detect nitrite due to their simplicity to use, low cost, portability, sensitivity, and fast measurement times without requiring experienced personnel.^18,19^

Among the electrodes, glassy carbon electrodes are the most widely used due to their high chemical stability and large polarization window.^20–22^ On the other hand, screen-printed electrodes (SPEs) based on carbon offer advantages such as potential for miniaturization, and low sample requirements compared to bulky electrodes.^23^ Nevertheless, the fabrication procedures for SPEs are time-consuming, with multiple steps,^24^ and require complex procedures for ink formulations.^25^ In this regard, LIG offers straightforward patterning of graphene-based surfaces in ambient air without any inks and at the same time overcomes the disadvantages associated with the conventional methods for the synthesis of graphene.^26,27^ LIG consists of a porous multilayer graphene bonded with sp^2^-bonded carbon atoms and provides high conductivity and surface area for electrochemical sensors^28^ and there have been several LIG-based electrochemical sensors to detect targets with good sensing properties, reproducibility, and stability.^29,30^

Concerning the detection of nitrite, gold (Au),^31^ silver (Ag),^32^ copper (Cu),^33^ and cobalt (Co) nanoparticles (NPs) have been investigated by combining with different carbon nanomaterials.^12,34^ The reason is that only carbon materials suffer from poor electrocatalytic activity.^35^ For example, Chu *et al.*^36^ modified the glassy carbon electrode by successive deposition of reduced graphene oxide and electrodeposition of copper nano dendrites to detect nitrite with a LoD of 0.06 μM. Further, a GCE modified with silver-reduced graphene oxide was used to quantify nitrite.^37^ LIG-based electrodes modified with CNT and Au NPs,^38^ polyvinyl chloride-based ion selective electrode,^30^ nanocarbon and AuNPs,^39^ and nitrogen and oxygen-doped LIG films^40^ were developed for the electrochemical detection of nitrite. On the other hand, Zhang *et al.*^35^ highlighted the potential of carbon fiber paper for detection, which was fabricated by implementing multiple steps that involved heating at high temperatures and drying.

In this research investigation, we report on the use of novel carbon fibers, termed LIF, in combination with CuPc (CuPc-LIF/LIG) as a novel modification material for LIG electrodes. The enhancement in the electrochemical performance after modification by LIF and the electrocatalytic activity achieved through CuPc towards the oxidation of nitrite was investigated by cyclic voltammetry and electrochemical impedance spectroscopy (EIS). The detection properties of the CuPc-LIF/LIG electrode that include LoD, linear range, repeatability, reproducibility, and shelf life of the developed electrode were evaluated. The analytical capability of the sensor was tested by the detection of nitrite in ground, tap, and mineral water. The performed investigations and obtained results in this work show the potential application of LIF as a modifier material for electrochemical sensors, and also demonstrate the good nitrite detection capability of the CuPc-LIF/LIG electrode.

**Fig. 1:**
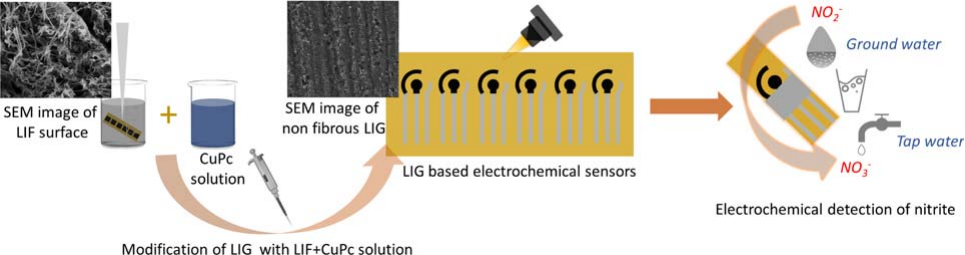
Graphical illustration of (a) the sonication of LIF electrodes for the dispersion and preparation of the composite of LIF and CuPc, (b) fabrication of LIG electrodes by CO_2_ laser and modification with the composite by drop-casting on the surface, and (c) electrochemical detection of nitrite in ground, drinking, and tap water.

Fig. 1 shows the graphical illustration of (a) the preparation of LIF dispersion by ultrasonication, (b) the schematic of the fabricated LIG electrode and its modification with the CuPc-LIF composite, and (c) the electrochemical detection of nitrite in ground, drinking, and tap water. Further details on materials and preparation of dispersion and electrodes, including the measurement parameters, are elaborated in the Materials and methods section and the dimensions for the electrodes are shown in Fig. S1.†

## Materials and methods

### Materials used

All the reagents used in this work were of analytical grade and used without any further purification. Sodium nitrite (NaNO_2_), sodium phosphate dibasic heptahydrate and monobasic monohydrate (Na_2_HPO_4_·7H_2_O and NaH_2_PO_4_·H_2_O), sodium bicarbonate (NaHCO_3_), potassium carbonate (K_2_CO_3_), potassium nitrate (KNO_3_), magnesium chloride (MgCl_2_), copper sulfate (CuSO_4_), potassium acetate (CH_3_CO_2_K), sulfuric acid (H_2_SO_4_), sodium hydroxide (NaOH) and potassium ferri-ferrocyanide were purchased from Merck, Germany. A Kapton sheet of thickness 110 μm was purchased from Dupont, Germany.

### Fabrication of LIG electrodes

The LIG electrodes were fabricated by a computer-controlled CO_2_ laser system (Epilog Mini 24) with a wavelength of 10.6 μm from Epilog. The three-electrode pattern was generated in the computer with control over the laser parameters to produce LIG surfaces. Specifically, to realize the three-electrode LIG void of fibers, a laser power of 4 W, scan rate of 50 mm s^−1^, and 1200 DPI were selected. The detailed procedure for fabricating the electrochemical LIG-based electrodes is as follows. Initially, the Kapton film was covered with a mask and left overnight to ensure good adhesion. The next step involved laser-cutting the mask at prescribed locations to apply silver for the contacts and reference electrodes and leave it to dry before engraving LIG. Finally, the LIG-based working and counter electrodes were engraved to ensure the alignment with the realized contacts. An insulation tape was used as a passivation layer for the three-electrode system. For direct laser writing of LIF surfaces, the parameters used were 12 W, 125 mm s^−1^, and 1200 DPI.

### Preparation of LIF and CuPc composites

For the preparation of LIF dispersion, six LIF squares of 5 mm^2^ dimensions were immersed in the isopropanol solution. The solution was then ultrasonicated for 24 hours, at which point, through visual inspection, it was confirmed that the surface was completely free of superficially bound materials. 5 mg of CuPc was dispersed in an isopropanol solution and was bath sonicated for 20 minutes to homogenize the solution. To prepare the nanocomposite dispersion, LIF and CuPc solution were mixed in a 1 : 1 ratio and the solution was bath sonicated before subsequent use.

### Instrumentation and measurement methods

All the electrochemical measurements were performed by using a commercial potentiostat, PalmSens4 from EKTechnologies, Germany. The EIS measurements of the electrodes in 5 mM [Fe(CN)_6_]^4−3−^ in 0.1 M KCl solution were performed from 50 000 Hz to 1 Hz at a dc potential of 0.2 V. The spectra were fit by using PS Trace software provided by PalmSens. The analysis of electrode behavior towards nitrite was performed by adjusting the pH of the solution by H_2_SO_4_ and NaOH. The detection by square wave voltammetry was performed from 0.2 to 1 V at a frequency of 10 Hz. FTIR analysis of the surfaces was performed by using an INVENIO S spectrometer from Bruker, Germany, in attenuated total reflectance mode, and the software compensated for the influence of the environment. Raman measurements were obtained by using a Raman plus 532H laser from Metrohm, Germany, with an integration time of 60 seconds and a maximum power of 30 mW for spectra acquisition. The measurements were obtained at three different positions on the surface for all the electrodes. SEM imaging was performed by using a Nova NanoSEM 200 microscope.

## Results and discussion

### Characterization of LIF

**Fig. 2:**
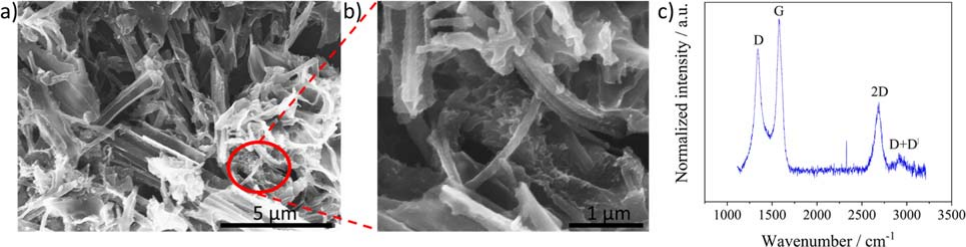
(a) SEM images of LIG nanostructures drop coated on an ITO substrate, (b) magnified image highlighting the fibers, and (c) average of the Raman spectra obtained at three different positions.

Fig. 2 shows the scanning electron microscopy (SEM) images and Raman spectra of the dispersions of LIG fibers drop-coated on an indium tin oxide substrate and Fig. S2† shows the image of the LIF surface before immersion and sonication. The nanostructures in Fig. 2 are identified by sheet and fiber-like structures similar to the morphology of the LIF surface in Fig. S2.† The magnified images in Fig. 2b show that the fibers are intertwined as bundles and wrapped around the sheet structures. The identified sheet morphology has been hypothesized to fragment into fibrous morphologies^9^ with the rise in power. Notably, the coexistence of the sheets, fibers, and subsequent fragmentation into droplets in the dispersion was also observed in this work. However, as the fibers were evident all around the sheet structures, the terminology for describing the solution and the modified electrode was LIF.

Fig. 2c shows the Raman spectra comprising bands at 1338 cm^−1^, 1572 cm^−1^, 2684 cm^−1^, and 2916 cm^−1^ that are assigned to D, G, 2D, and D + D^I^ bands, respectively. The presence of D band indicates the surface is induced with disorders and is typically active when defects are present in the sample. The G band indicates sp^2^-bonded carbon originating from first order scattering processes. The 2D band is an overtone of the D band and is the prominent band in graphene surfaces and the D + D^I^ band is related to two phonon defect-induced activity.^41–43^ The ratio of the D to G bands, a representative of the defect density, was quantified from the average of three spectra obtained at distinct positions, and its value was 0.82 ± 0.01, indicating the defect-induced nature of the LIG nanostructures.

### Characterization of LIF-modified LIG surfaces

**Fig. 3:**
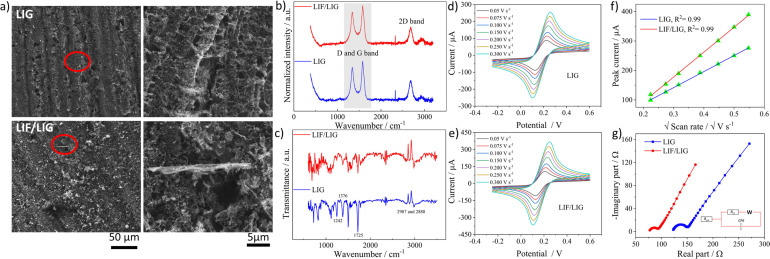
(a) SEM images of bare LIG and LIF/LIG and to the right are corresponding magnified images, (b) and (c) Raman and FTIR spectra of LIG and LIF/LIG, respectively, (d) and (e) the CV curves obtained in 5 mM [Fe(CN)_6_]^4−3−^ at different scan rates for LIG and LIF/LIG, respectively, (f) the plot of peak currents *versus* square root of scan rate and (g) the EIS spectra of the electrodes with the inset showing the equivalent circuit used for fitting.

The as-prepared dispersion of LIF was used to modify the working electrode of the LIG. SEM images shown in Fig. 3a depict a highly porous network of LIG with sizes of the pores in the micrometer range that are formed due to the release of gaseous products.^44^ The magnified image depicts the interconnected texture of the porous LIG that provides the high surface area needed for electrochemical applications. The SEM image of the LIF/LIG shown in Fig. 3a is characterized by the random distribution of fibrous bundles of varied sizes on LIG and the magnified image reveals one such fiber bundle. Notably, the drop cast fibers are adsorbed and intertwined within the porous interconnected network, which enables the achievement of the desired sensor properties, including good stability and reproducibility, as discussed below. Fig. 3b shows the average Raman spectra of the LIG and LIF/LIG electrodes obtained from three different positions on the surface. Both the electrodes have similar Raman spectra with characteristic D, G, and 2D bands. As LIG is a highly defect-induced surface,^45^ a notable difference in the *I*_D_/*I*_G_ ratio was not observed after modification with LIF and the quantified values were 0.80 ± 0.02 for LIG and 0.815 ± 0.05 for LIF/LIG electrodes. In addition, the *I*_2D_/*I*_G_ ratio is sensitive to the layers and its value rises with a decrease in the number of layers. The quantified ratio is 0.51 ± 0.01 for the LIG surface, indicating the multilayer nature of graphene.^46^ The functional groups of the surfaces were confirmed by FTIR spectroscopy and are shown in Fig. 3c. LIG and LIF/LIG surfaces consist of bands at 2987 cm^−1^ and 2880 cm^−1^, 1725 cm^−1^, 1610 cm^−1^, 1376 cm^−1^, and 1242 cm^−1^ that are associated with vibrations from stretching of C–H bonds, C=O in the carbonyl group, C=C in the graphene structures, C–O–C from the epoxide group and C–O stretching, respectively.^47–49^

The electrochemical behavior of both the electrodes was investigated by recording cyclic voltammetry (CV) curves at different scan rates in a 5 mM of ferri-ferrocyanide ([Fe(CN)_6_]^4−3−^) in 0.1 M potassium chloride solution as shown in Fig. 3d for LIG and Fig. 3e for LIF/LIG electrodes. As can be seen from the figures, after the functionalization with LIF, an increase in peak current and a reduction in peak potential difference were observed as compared to the bare LIG. This confirms that the modification by LIF enhances the electrochemical activity and conductivity of the electrode. Fig. 3f depicts the plot of peak current as a function of the square root of the scan rate. A linear increase in the values of peak currents was observed in good *R*^2^ values for both electrodes, suggesting diffusion as the dominant mechanism. The evaluation of the active surface area and heterogeneous electron transfer rate provides a quantitative estimate of the enhancement resulting from the modification with LIF. The active surface area of both the electrodes was evaluated using the Randles–Sevcik equation for a quasi-reversible reaction^50^ as shown in the below equation:1
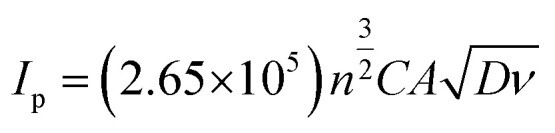


*I*_p_ is the peak current, *n* denotes the electrons transferred, *D* is the diffusion coefficient of potassium ferricyanide and the value is given as 7.6 × 10^−6^ cm^2^ s^−1^, *A* is the surface area and *ν* is the scan rate (V s^−1^). By evaluating the peak currents for both electrodes at a scan rate of 0.1 V s^−1^, the active surface area was calculated to be 0.669 and 0.837 cm^2^ for LIG and LIF/LIG electrodes, respectively, indicating the high surface area of the LIF on the surface. The heterogeneous transfer rate constant was calculated according to eqn (2)^51^ by the Nicholson method where the value of *ψ*, a kinetic parameter, is based on the peak potential difference of the oxidative and reductive currents evaluated from CV. *D*_O_ and *D*_R_ denote the diffusion coefficients for ferricyanide (7.6 × 10^−6^ cm^2^ s^−1^) and ferrocyanide (6.3 × 10^−6^ cm^2^ s^−1^), *F* is the Faraday constant (96 485 C), *R* is the universal gas constant (8.314 J K mol^−1^), *T* is the absolute temperature in *K* and *α* is the charge transfer coefficient and its value is 0.5.2
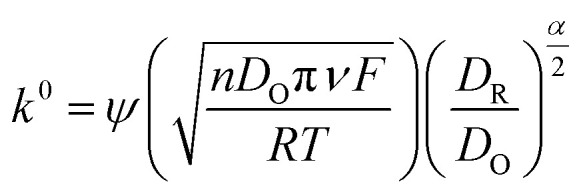


The *k*^0^ values were obtained from the procedure mentioned in ref. 52 and were found to be 0.025 and 0.0343 cm s^−1^ for LIG and LIF/LIG, respectively, which indicates the enhanced electrochemical activity of the electrode after modification with LIF. The charge transfer characteristics were further investigated by performing EIS in [Fe(CN)_6_]^4−3−^ solution, which has a semicircle part at high frequencies and a diffusion part at low frequencies as seen in Fig. 3g. The data was fit using the Randles equivalent circuit shown in the inset of Fig. 3g, where *R*_sol_ and *R*_ct_ are the solution resistance and charge transfer resistance, respectively, CPE is the constant phase element and W is the Warburg impedance. The obtained *R*_ct_ values are 17.28 W and 8.88 W for LIG and LIF/LIG electrodes, respectively, which complement the results from CV and confirm the conductive nature and high electrochemical activity achieved by the modification with LIF on LIG.

### Optical and electrochemical characterization of CuPc-LIF/LIG

**Fig. 4:**
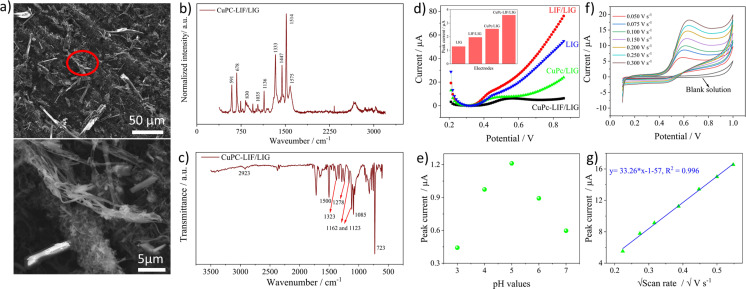
(a) SEM image of the CuPc-LIF/LIG electrode with the magnified image at the bottom, (b) and (c) Raman and FTIR spectra of CuPc-LIF/LIG electrodes, respectively, (d) the SWV response of LIG, LIF/LIG, CuPc/LIG, and CuPc-LIF/LIG modified electrodes towards 100 μM concentration of nitrite at pH5 with the inset showing the bar graph of evaluated peak currents, (e) the peak currents recorded from SWV curves at different values of pH for 0.1 M PBS with nitrite, (f) the CV curves of the CuPc-LIF/LIG electrode at different scan rates in 500 μM solution of nitrite at pH 5 and (g) the corresponding plot of peak current *versus* square root of scan rate.

For the electrochemical detection of nitrite, the surface was modified with the composite of CuPc-LIF solution. CuPc was used as it is known to have good catalytic behavior towards the oxidation of nitrite. Fig. 4a shows the SEM images of CuPc-LIF-modified LIG (CuPc-LIF/LIG) wherein rod-like structures of various dimensions typical of CuPc molecules^53,54^ were identified. The magnified image shows the fiber structures surrounding the CuPc molecules of smaller sizes. Raman spectra with CuPc on the surface were distinctive with new peaks originating in the molecule's spectra as seen in Fig. 4b. The band at 591 cm^−1^ is attributed to the benzene ring deformation of CuPc and those at 678 cm^−1^ and 830 cm^−1^ are related to C–N–C stretching vibrations.^54^ The C–H bending vibrations were noticeable at 1035 cm^−1^ and 1136 cm^−1^.^53^ The spectral range from 1350 to 1550 cm^−1^ is the fingerprint for different phthalocyanine molecules. For CuPc, the bands identified at 1333 cm^−1^, 1447 cm^−1^, and 1514 cm^−1^ correspond to pyrrole stretching, isoindole stretching, and the C–N–C bridge bond linked to the copper ion of the molecule.^55^ The FTIR spectra shown in Fig. 4c also consist of new bands originating from the CuPc molecules on the surface. The bands at 2923 cm^−1^ and 1085 cm^−1^ are assigned to C–H stretching and bending vibrations, respectively, and the band at 1500 cm^−1^ is related to pyrrole deformations. The vibrational mode of C=N–C is visible at 1323 and 1278 cm^−1^ and is related to Cu–N bonding.^56^ Finally, the highly intense and sharp band at 723 cm^−1^ indicates the alpha crystalline phase of CuPc.^57,58^ The electrocatalytic activity of the CuPc-LIF/LIG electrode towards nitrite was investigated by recording SWV curves in a nitrite solution of 100 μM concentration as shown in Fig. 4d and the inset shows the bar graphs of evaluated peak currents for different electrodes. The high electrocatalytic activity of CuPc molecules was indicated by higher currents recorded on CuPc/LIG as compared to the LIF/LIG electrode. Further, LIF/LIG had better currents compared to bare LIG towards nitrite oxidation following the results from the redox couple. The LIF and CuPc composite modified LIG demonstrated the best response for the oxidation of nitrite, as seen from the bar graph of peak currents in the inset of Fig. 4d, confirming the synergetic effect of LIF and CuPc towards the oxidation of nitrite. Fig. 4e shows the peak currents from SWV at different pH values (3 to 7) in phosphate buffer solution (PBS) containing 50 μM nitrite concentration. pH 5 shows the maximum currents for the oxidation of nitrite. It is well known that nitrite is unstable in acidic media and gets converted to nitrate; thus, lower currents were recorded at pH 3 and 4. On the other hand, protonation is an important aspect in the oxidation of nitrite, and the shortage of protons at a pH greater than five leads to low currents.^59,60^ Before further investigations, the response towards oxidation of nitrite with different amounts of CuPc-LIF on LIG electrodes was investigated. 3 μl was found to be optimal as high currents were recorded and the relevant data is presented in Fig. S3 of ESI.†

Fig. 4f shows the CV curves recorded at different scan rates in 500 μM nitrite concentration at pH 5. The CV of the blank solution does not have any peaks in the selected potential window. Upon the addition of nitrite, oxidation peaks, and their peak values increasing with scan rate were observed. The plot of peak current *versus* square root of scan rate shown in Fig. 4g had a linear correlation with an *R*^2^ value of 0.996, concluding the fact that the dynamics of the reaction is based on diffusion. The widely accepted reaction mechanism^61^ is the electrooxidation of nitrite to nitrate involving two electrons, as shown in the below equation.

3 NO_2_^−^ + H_2_O → NO_3_^−^ + 2H^+^ + 2e^−^

### Electrochemical detection of nitrite by the CuPc-LIF/LIG electrode

**Fig. 5:**
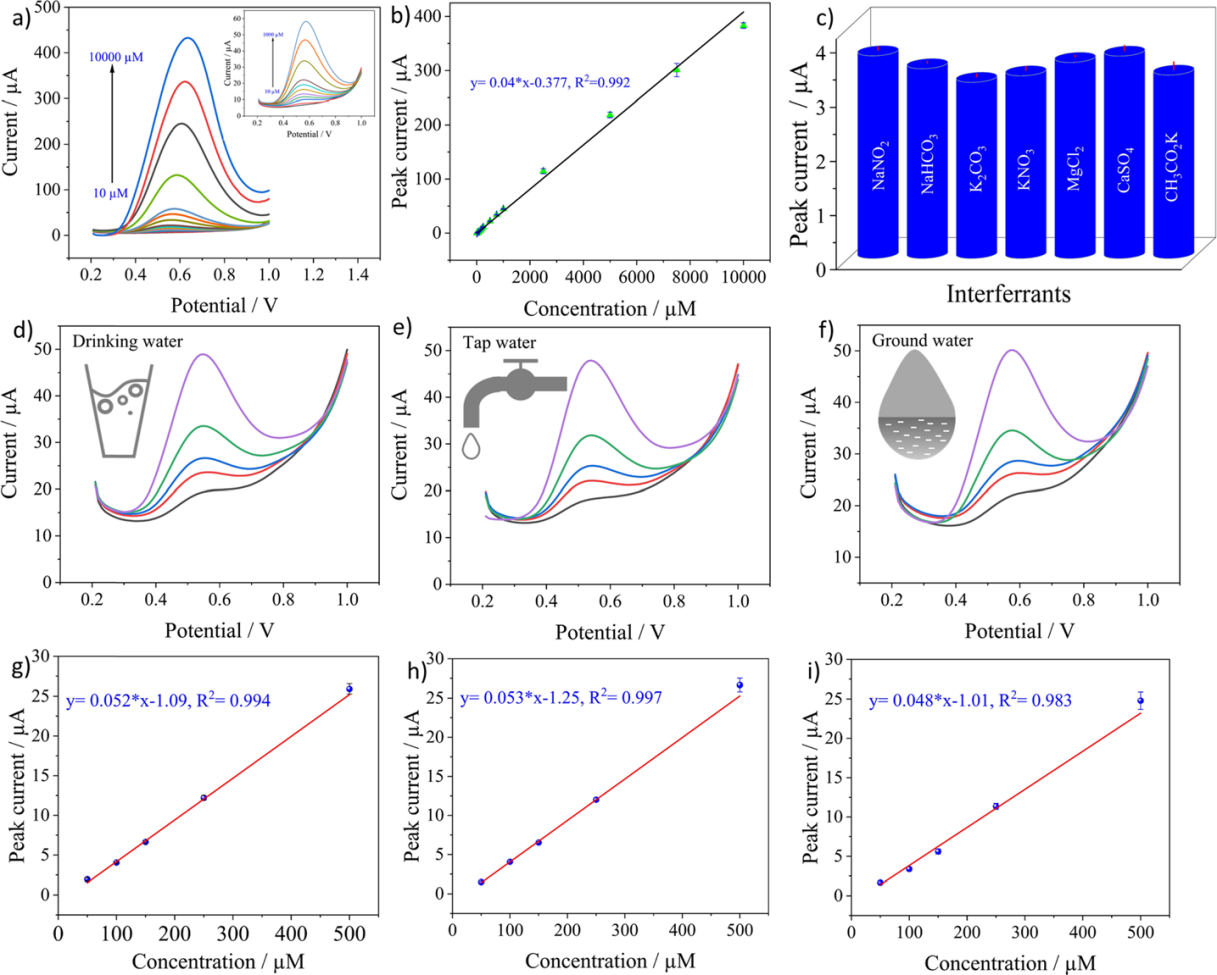
(a) Electrochemical detection of nitrite from 10 to 10 000 μM and the inset shows the curves from 10 to 1000 μM, (b) the calibration curve, (c) selectivity studies by plotting the peak currents of 100 μM of nitrite with 1000 μM concentration of interfering substances, detection of 50, 100, 150, 250, and 500 μM spiked concentrations of nitrite in (d) drinking water, (e) tap water, and (f) groundwater, and (g), (h), and (i) the calibration curves for drinking, tap and ground water respectively.

The analytical performance of the sensor was evaluated by recording SWV curves in different concentrations of nitrite prepared in 0.1 M PBS solution at pH 5 as seen in Fig. 5a. The inset shows the magnified image of the recorded SWV curves at low concentrations from 10 to 1000 μM. It can be observed that the oxidation currents increase with the concentrations. The calibration curve in Fig. 5b was plotted by obtaining the average of three measurements for a particular concentration with its standard deviation. The plot shows a linear relationship across the entire concentration range from 10 to 10 000 μM with 99.06% as the coefficient of determination, indicating an excellent linear fit of the data. LoD was calculated from the equation below.4
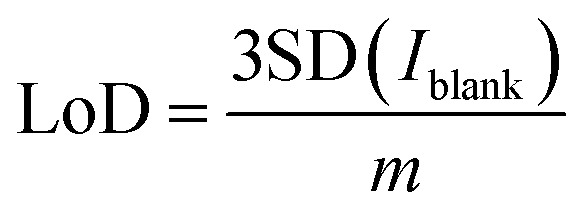


SD is the standard deviation of current recorded in the blank solution and *m* signifies the slope of the regression line. The quantified LoD was 0.162 μM with a linear range from 10 to 10 000 μM. The good sensitivity achieved by the electrode can be related to the high catalytic activity of CuPc^62^ towards nitrite and also the high electrochemical activity offered by LIF on LIG. The lack of redox activity of metal centers in CuPc molecules implies that ring based processes are dominant^63^ and the nitrite ions bind to the ligand of the molecule.^64^ After the binding, the high electron transfer rate offered by LIF on LIG creates an appropriate medium for the electron transfer to occur with the electrode for the oxidation of nitrite.

The selectivity of the CuPc-LIF/LIG electrode towards nitrite was evaluated by recording the oxidation currents in the presence of 10-fold concentrations of common cationic and anionic interferants in the buffer solution. Fig. 5c shows the peak current data for nitrite oxidation in the presence of NaHCO_3_, K_2_CO_3_, KNO_3_, MgCl_2_, CaSO_4_, and CH_3_CO_2_K. The electrode showed almost negligible interference from other ions with a maximum relative error percentage of 12.11% for nitrate. The results indicate the sensor's good selectivity with a potential for detecting nitrite in real samples. The reason for the good selectivity of the electrode towards nitrite could be attributed to the potential window of SWV, which is specific to the oxidation of nitrite. In addition, the high binding efficiency of nitrite to CuPc as explained in other works^65^ led to the high selectivity.

Testing in aqueous environments is crucial to evaluate the practical applicability of the electrochemical sensor. In this regard, the credibility of the developed sensor was tested in tap, mineral, and groundwater by the standard addition method directly without any further purification or filtration of the matrix. The sensors were tested at different spiked concentrations of nitrite ranging from 50 to 500 μM. Fig. 5d–f show the SWV curves recorded at 50, 100, 150, 250 and 500 μM nitrite concentrations for the three real samples. Triplicate measurements were obtained for a single concentration and their SD was included in the calibration curves. The electrode showed a linear response for all three real samples with *R*^2^ values of 0.994, 0.997, and 0.983 for mineral, tap, and groundwater, respectively, as seen in Fig. 5g–i. Based on the peak currents recorded for these concentrations, the sensor showed a good recovery, with values not exceeding 11% for all the concentrations, which can be seen in Table S1 in ESI.† Further investigation on the applicability of the electrochemical sensor is performed by the matrix effect percentage from the below equation:^66^5
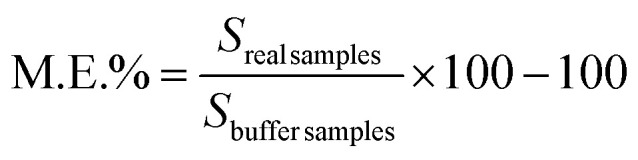
where M. E% stands for the matrix effect, *S*_real samples_ is the slope of the calibration curves of real samples, and *S*_buffer samples_ is the slope of the calibration curve in the buffer solution. The slope of the calibration curve from the buffer solution was extracted by considering only the concentrations used for real sample analysis as shown in Fig. S4.† The obtained values are 10.63% for mineral water, 12.76% for tap water, and 2.12% for groundwater. The low values of M.E.% combined with good recoveries in the real matrices indicate an excellent analytical capability of the sensor in real samples. Table 1 shows the comparison of the sensor's performance with previous studies that have elaborated laser-based electrode fabrication or copper phthalocyanines as the functionalization material for nitrite detection. The proposed sensor in this work displayed comparable or significantly better properties in either obtained linear range or LoD for the detection of nitrite. The obtained sensor properties also indicate the potential advantages of using LIF as a functionalization material for electrochemical sensors.

**Table 1:** Comparison of sensing performance of different laser-based electrochemical sensors for the detection of nitrite

Electrode material	Method	Linear range (μM)	LoD (μM)	Shelf life	Ref.
*CuPc-LIF/LIG*	*Voltammetry*	*10 to 10 000*	*0.162*	*30 days*	*This work*
Nitrite ionophore/LIG	Potentiometry	10 to 100 000	4.83	—	30
Ag NPS/graphitic carbon	Voltammetry	1 to 600, 600 to 4000	0.117	30 days	67
Au/NIO/Rh/LIG	Voltammetry	1 to 1000	0.3	15 days	68
CuPc/GCE	Voltammetry	100 to 1000	0.67	—	69
N, O doped LIG	Voltammetry	5 to 450	0.8	—	40
LRGO/ION-RGO	Voltammetry	10 to 400	7.2	—	70
Laser pyrolyzed sensors	Voltammetry	40 to 250	4.3	15 days	71
F-MWCNT-AUNPS/LIG	Voltammetry	10 to 140	0.9	—	38
CS/NP-LIG	Voltammetry	0.15 to 15	0.018	15 days	72
CuTSPc/PLL/GCE	Amperometry	0.12–12	0.036	30 days	73
Chit-TsCuPc/GCE	Amperometry	0.005–0.065 nM	0.022	30 days	74

### Repeatability, reproducibility, and stability studies

**Fig. 6:**
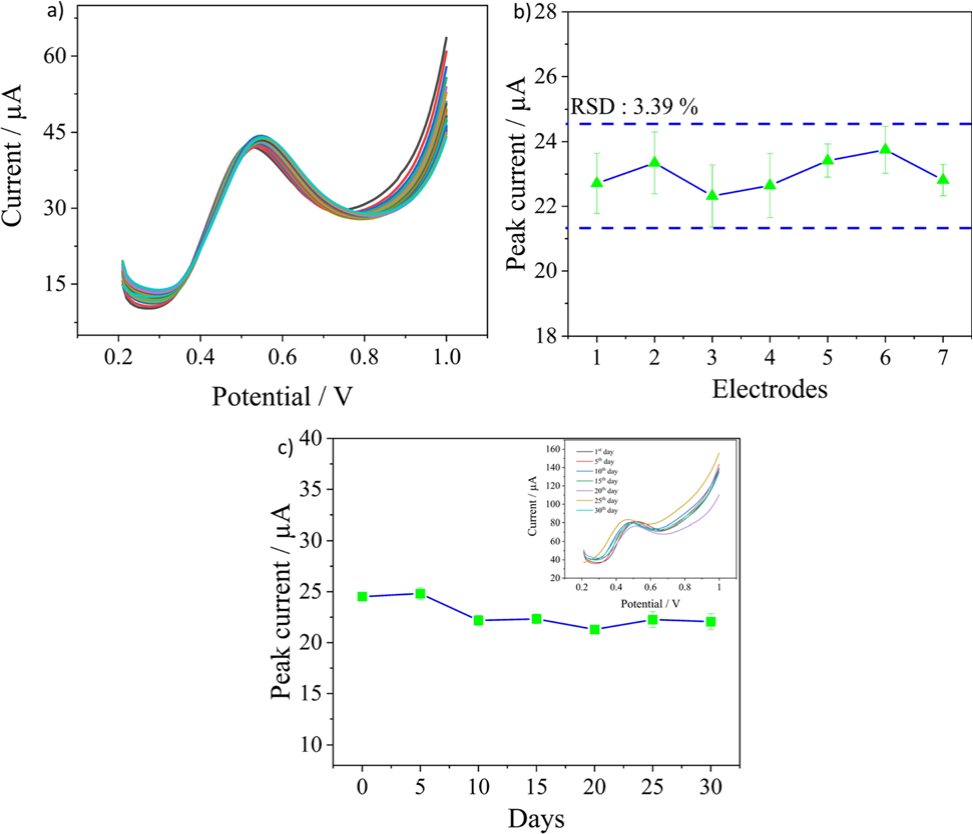
(a) 30 measurements on a single sensor for repeatability analysis with the inset showing the recorded peak currents, (b) the peak current for seven fabricated sensors and (c) the long-term stability analysis by recording peak current as a function of days and the inset shows the SWV curves.

The other important parameters to ascertain the sensor's applicability in practical scenarios are its reproducibility, repeatability, and stability and they were evaluated in 0.1 M PBS solution containing a nitrite concentration of 500 μM. The repeatability of the sensor was evaluated by recording 30 consecutive measurements on the same electrode. As observed in Fig. 6a, the sensor had excellent repeatability as the peak currents were almost similar with a very low RSD value of 1.64%. The reproducibility of the sensor was investigated by measuring the peak currents obtained from three measurements each on seven different electrodes prepared under the same conditions. An RSD of 3.39% was calculated, as seen in Fig. 6b, which confirms the good quality in the fabrication procedure of electrodes leading to good reproducibility. The long-term stability of the electrodes was analysed by recording the peak currents over 30 days as seen in Fig. 6c. The tests were performed once every five days, and three measurements were obtained for every test in the same concentration. At the end of the 30th day, around 86.2% of the current signal was still retained, demonstrating good stability for the sensors. Overall, the developed sensor has excellent repeatability in measurements with good batch reproducibility and long-term stability as well.

## Conclusions

This work reports on the potential of LIF as a novel functionalization material for electrochemical sensors. Owing to the loosely bound nature of fibers on the surface of the substrate after engraving by laser, we proposed to use ultrasonication for the extraction of LIF in the form of a dispersion. The modification of the LIG electrode with LIF dispersion enhanced the active surface area and electron transfer rate, thus indicating its potential as a functionalization material.

For the detection of nitrite, the surface of LIG was modified with the CuPc-LIF composite, which demonstrated a good catalytic effect towards the oxidation of nitrite. A good LoD of 0.162 μM and a large linear range from 10 to 10 000 μM were achieved, surpassing the previous works that have used laser for fabrication of electrodes or CuPc as the functionalization material. In addition, the developed sensor demonstrated excellent reproducibility and repeatability with good recoveries and minimal matrix effect for the detection in mineral, tap, and groundwater.

With this study, we could prove a very good potential of LIF as a modification material to realize electrochemical sensors for the detection of nitrite. The next step in this direction would be to investigate the type of structures, role of aspect ratio of the fibers and the quality of the obtained dispersion by optimizing the choice of laser parameters for engraving the LIF surfaces. This would potentially imply that fibers with tunable dimensions, and physical, electrical, and chemical properties can be realized. This work builds therefore a solid basis for new research directions for cost-effective and simple fabrication of customizable LIF and their exploitation in different applications. Further, the developed sensor in this work based on CuPc-LIF/LIG has the desired properties and potential to be implemented in practical scenarios to monitor the concentrations of nitrite.

## Data availability

The data can be obtained from the corresponding author upon request.

## Author contributions

Conceptualization: Anurag Adiraju, Olfa Kanoun; data curation: Anurag Adiraju, Aditya Jalasutram; formal analysis: Anurag Adiraju, Aditya Jalasutram, Ammar Al-Hamry, Malak Talbi, Junfei Wang; supervision: Anurag Adiraju, Olfa Kanoun; funding acquisition: Olfa Kanoun, Ammar Al-Hamry; methodology: Anurag Adiraju, Aditya Jalasutram, Ammar Al-Hamry; investigation: Anurag Adiraju, Aditya Jalasutram, Malak Talbi, Junfei Wang; resources: Olfa Kanoun, Christoph Tegenkamp; visualization: Anurag Adiraju, Aditya Jalasutram; writing – original draft: Anurag Adiraju, Malak Talbi; writing – review and editing: Olfa Kanoun, Christoph Tegenkamp.

## Conflicts of interest

There are no conflicts to declare.

## Supplementary Material

RA-014-D4RA03341H-s001
