# Supplementary material for: Laser-induced fibers and copper phthalocyanine modified laser-induced graphene electrodes for sensitive and selective electrochemical detection of nitrite
Source: RSC Adv. 2024 Sep 9;14(39):28648–58. doi: 10.1039/d4ra03341h (PMC11381946; doi:10.1039/d4ra03341h)
Supplement: RA-014-D4RA03341H-s001 [file RA-014-D4RA03341H-s001.pdf]

Supplementary Information (SI) for RSC Advances.
This journal is © The Royal Society of Chemistry 2024

Supplementary information

Laser induced fibers and copper-phthalocyanine modified laser induced graphene electrodes for sensitive and selective electrochemical detection of nitrite

Anurag Adiraju,*[a] Aditya Jalasutram[a] , Ammar Al-Hamry[a], Malak Talbi[a], Junfei Wang[a], Christoph Tegenkamp[b], Olfa Kanoun[a]

E-mail: adiraju.anurag@etit.tu-chemnitz.de

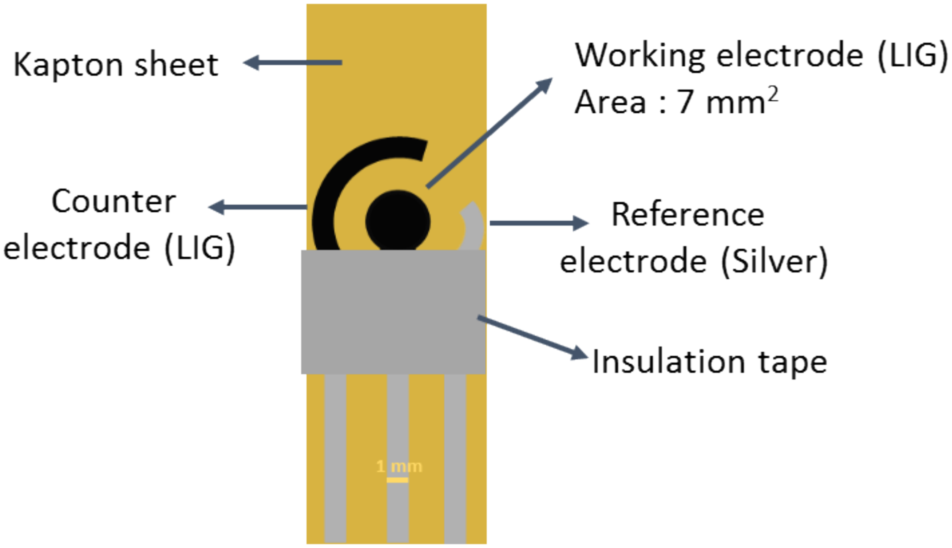

Figure S1. Pictorial representation of the three electrode LIG electrochemical sensor including the dimensions

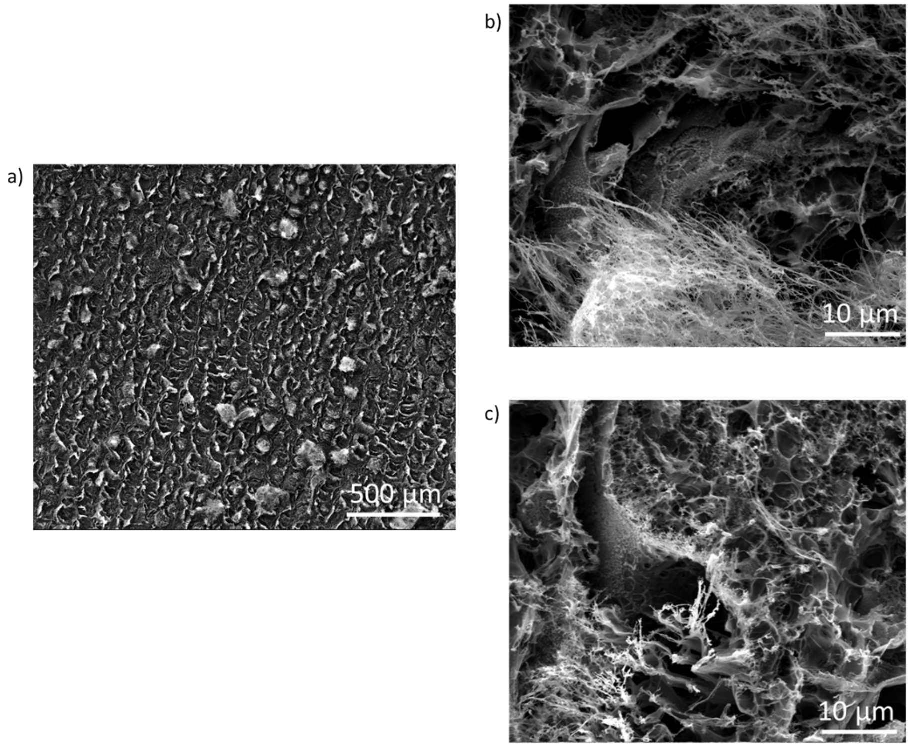

FigureS2. SEM images of a) LIGF surface engraved at 12W, magnified images of b) sheet structures and c) fibrous morphology on the surface

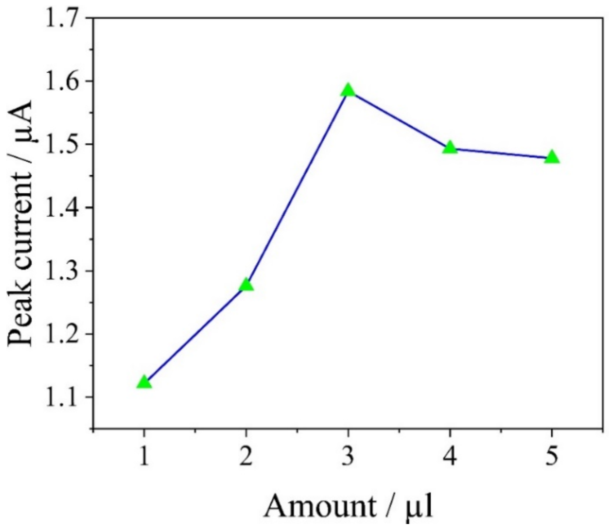

Figure S3. Variation in peak current obtained from SVW at 50µM nitrite concentration as function of amount of CuPc/LIGF on the electrode

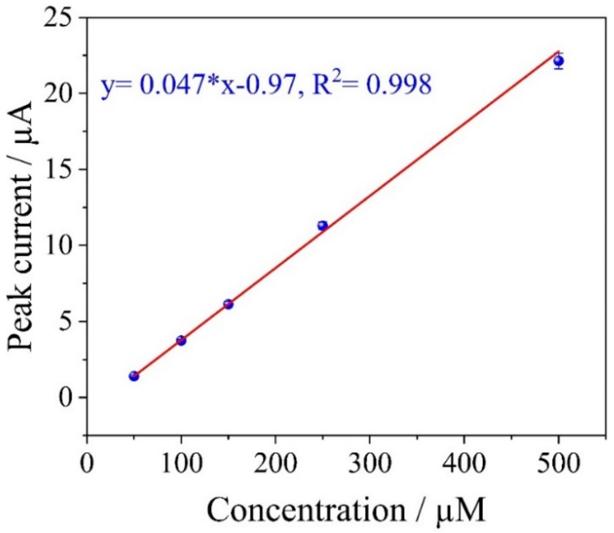

Figure S4. Truncated calibration curves of nitrite detection in buffer solution obtained from the concentrations used for analysis in real samples

Table S1: The calculated recoveries of CuPc/LIGF/LIG electrode at different concentrations in drinking, tap, and ground water

Spiked	found (DW) μM	recovery (%)	found (TW) μM	recovery (%)	found (GW) μM	recovery (%)
50	53.26	106.5	48.24	96.4	54.01	108.2
100	104.38	104.3	105.12	105.1	90.72	90.7
150	154.2	102.8	158.02	105.3	138.59	92.3
250	264.1	105.6	275.23	110.09	257.01	102.8
500	532.6	106.5	552.83	110.5	536.19	107.2

*DW: Drinking water, TW: Tap water, GW: groundwater
